# Exposure to Subclinical Doses of Fumonisins, Deoxynivalenol, and Zearalenone Affects Immune Response, Amino Acid Digestibility, and Intestinal Morphology in Broiler Chickens

**DOI:** 10.3390/toxins17010016

**Published:** 2025-01-01

**Authors:** Revathi Shanmugasundaram, Laharika Kappari, Mohammad Pilewar, Matthew K. Jones, Oluyinka A. Olukosi, Anthony Pokoo-Aikins, Todd J. Applegate, Anthony E. Glenn

**Affiliations:** 1 U. S. National Poultry Research Center, Agriculture Research Service, U.S. Department of Agriculture, Athens, GA 30605, USA; 2 Department of Poultry Science, University of Georgia, Athens, GA 30602, USA; 3 Southern Poultry Research Group, Inc., Watkinsville, GA 30677, USA

**Keywords:** mycotoxins, broilers, immune response, intestinal health, amino acid digestibility

## Abstract

Fusarium mycotoxins often co-occur in broiler feed, and their presence negatively impacts health even at subclinical concentrations, so there is a need to identify the concentrations of these toxins that do not adversely affect chickens health and performance. The study was conducted to evaluate the least toxic effects of combined mycotoxins fumonisins (FUM), deoxynivalenol (DON), and zearalenone (ZEA) on the production performance, immune response, intestinal morphology, and nutrient digestibility of broiler chickens. A total of 960 one-day-old broilers were distributed into eight dietary treatments: T1 (Control); T2: 33.0 FUM + 3.0 DON + 0.8 ZEA; T3: 14.0 FUM + 3.5 DON + 0.7 ZEA; T4: 26.0 FUM + 1.0 DON + 0.2 ZEA; T5: 7.7 FUM + 0.4 DON + 0.1 ZEA; T6: 3.6 FUM + 2.5 DON + 0.9 ZEA; T7: 0.8 FUM + 1.0 DON + 0.3 ZEA; T8: 1.0 FUM + 0.5 DON + 0.1 ZEA, all in mg/kg diet. The results showed that exposure to higher mycotoxin concentrations, T2 and T3, had significantly reduced body weight gain (BWG) by 17% on d35 (*p* < 0.05). The T2, T3, and T4 groups had a significant decrease in villi length in the jejunum and ileum (*p* < 0.05) and disruption of tight junction proteins, occludin, and claudin-4 (*p* < 0.05). Higher mycotoxin groups T2 to T6 had a reduction in the digestibility of amino acids methionine (*p* < 0.05), aspartate (*p* < 0.05), and serine (*p* < 0.05); a reduction in CD4+, CD8+ T-cell populations (*p* < 0.05) and an increase in T regulatory cell percentages in the spleen (*p* < 0.05); a decrease in splenic macrophage nitric oxide production and total IgA production (*p* < 0.05); and upregulated cytochrome P450-1A1 and 1A4 gene expression (*p* < 0.05). Birds fed the lower mycotoxin concentration groups, T7 and T8, did not have a significant effect on performance, intestinal health, and immune responses, suggesting that these concentrations pose the least negative effects in broiler chickens. These findings are essential for developing acceptable thresholds for combined mycotoxin exposure and efficient feed management strategies to improve broiler performance.

## 1. Introduction

Mycotoxins are toxic secondary metabolites produced by fungi that often contaminate major poultry feed ingredients (e.g., corn kernels), posing significant challenges to the poultry industry. These toxins are ubiquitously present in poultry house environments, with climate change and storage conditions further exacerbating contamination in animal feed commodities [1]. These toxic secondary metabolites are predominantly produced by fungi such as *Aspergillus*, *Penicillium*, and *Fusarium* species [2]. Key species such as *F. verticillioides* and *F. proliferatum* are known to contaminate corn and produce fumonisins (FB1, FB2, and FB3) [3], while *F. graminearum* and *F. culmorum* are known to produce deoxynivalenol (DON) and zearalenone (ZEA) [4]. According to DSM-Firmenich mycotoxin survey reports, 88% of the tested corn and corn by-products in the USA are contaminated with at least one mycotoxin, and 92% of the finished diet contains more than one mycotoxin [5,6]. Among these, FUM and DON are the most frequently detected in more than 70% of North American corn samples, with an average of 2589 and 1220 µg/kg, respectively. With corn being an integral part of poultry feed in the USA, the annual economic losses resulting from FUM, DON, and ZEA are estimated to be between USD 0.5 and 1.5 billion per year [7,8].

In the past, chickens were believed to be relatively resistant to mycotoxins compared to other poultry species [9]. However, due to the modern genetic selection of broiler chickens for rapid growth and high meat yield, their sensitivity to mycotoxins has increased. Although genetic pressure improved the production performance of chickens, they became more vulnerable to the negative effects of mycotoxins, compromising their immune system [10,11]. Despite established regulatory guidelines for individual mycotoxins by the European Food Safety Authority (EFSA) and the U.S. Food and Drug Administration (FDA) [12,13], the co-occurrence of multiple mycotoxins in poultry diet often leads to synergistic effects even at subclinical concentrations [14]. In the past, it was believed that a FUM concentration below 50 mg/kg feed and DON concentration below 5 mg/kg feed would not have negative effects on poultry [15,16].Previous studies were either conducted in chickens with a single mycotoxin [17,18] or with toxic doses of combined FUM and DON for a short period of time [19,20,21,22]. The presence of subclinical doses of multiple mycotoxins, such as FUM (3–20 mg/kg), DON (1–5 mg/kg), and ZEA (1 mg/kg), in feed decreases the tolerance level of each mycotoxin and negatively impacts poultry health by lowering body weight and nutrient digestibility. Furthermore, exposing chickens to a combined toxins, can disrupt the gut microbiota, damage the villi structure and gut health, exacerbate the liver and gut pathology, and increase susceptibility, as well as prolong recovery from diseases like salmonellosis, coccidiosis, and necrotic enteritis [19,20,23,24,25,26,27,28]. Our previous research identified that 3 mg/kg FUM and 4 mg/kg DON were found to be adequate to induce subclinical necrotic enteritis [24]. This suggests that mycotoxins at these levels could potentially affect the broiler’s overall health.

The effects of mycotoxins on animal system can be either immunostimulatory or immunosuppressive, depending on their chemical structure, concentration, and duration of exposure to toxins [29,30,31,32]. Mycotoxins, such as FUM and DON in poultry, contribute to immunosuppression by impairing the immune system’s ability to generate adequate immune responses, inhibiting cell proliferation, and reducing the phagocytic function of cells by altering the CD4:CD8 ratio [33,34,35,36,37]. This results in a decrease in antibody production and decreases disease resistance [10,24,25,38,39].

Chickens are continuous feeders; even subclinical doses of multiple mycotoxins can damage their intestines over time [14]. In poultry, the absorption of FUM and DON may vary between 1% and 6% [40]. This limited absorption of FUM and DON indicates that a substantial portion of non-absorbed toxin remains within the lumen of the gastrointestinal tract [40,41,42]. In addition, FUM and DON undergo entero-hepatic circulation, resulting in reabsorption and a prolonged retention time in the gut [43]. Long-term exposure to these mycotoxins is likely to damage the gut lining, increase gut permeability, and negatively affect gut health, immune response, and overall well-being in chickens. Depending on their bioavailability, FUM and DON also interfere with signaling pathways that regulate cell growth and apoptosis and suppress the immune response [26,29,44]. Though it is well accepted that mycotoxins can modulate immune responses [26,45,46,47], research on the effects of chronic exposure to subclinical doses of FUM and DON, particularly in relation to immune response and amino acid digestibility in broiler chickens, is limited. Since mycotoxins are known to cause immunosuppression, it is critical to understand the mechanisms and doses involved in compromising immune response and nutrient absorption. This knowledge is essential for developing strategies to mitigate the negative effects of mycotoxins and improve broiler performance. Therefore, the goal of the study was to determine the lowest mycotoxin combination that would not compromise immune function or production performance in broiler chickens. With the increasing incidence of the co-occurrence of mycotoxins in poultry feed and growing concerns related to subclinical impacts on immune health, the objective of this study was to investigate the different combinations of mycotoxins FUM, DON, and ZEA at low doses below FDA guidance level on the amino acid digestibility, immune responses, and intestinal morphology of broiler chickens.

## 2. Results

### 2.1. Effect of Combined Doses of Mycotoxins on Production Performance

A significant difference (*p* < 0.05) was observed in BWG among treatment groups on d21, d28, and d35 (Table 1). Birds in the T2 treatment, exposed to the higher concentration of mycotoxins, had a significantly lower BWG of 17% on d21 (*p* < 0.05), 13% on d28 (*p* < 0.05), and 12% on d35 (*p* < 0.05) than the control group. BWG in the T7 and T8 treatment groups was comparable to the control group on d21, d28, and d35. Birds in the T2 groups had a numerical increase in FCR by 11 points compared to the control group on d35 (*p* > 0.05).

### 2.2. Effect of Combined Doses of Mycotoxins on Apparent Ileal Amino Acid Digestibility

On d21, significant differences were observed in indispensable amino acids, such as methionine, and dispensable amino acids, like aspartate and serine (*p* < 0.05), as shown in Table 2. The T6 group had 9.6% lower methionine digestibility when compared to the control group T1 (*p* < 0.05). The T3 group had 27.3% lower aspartate and 23.4% lower serine digestibility when compared to the control group T1 (*p* < 0.05). There were no significant differences in the endogenous nitrogen, dry matter percentage, and ileal amino acid digestibility on d21 between treatment groups (*p* > 0.05).

### 2.3. Effect of Combined Doses of Mycotoxins on Jejunal and Ileal Histomorphology and Intestinal Lesion Score

**Figure 1:**
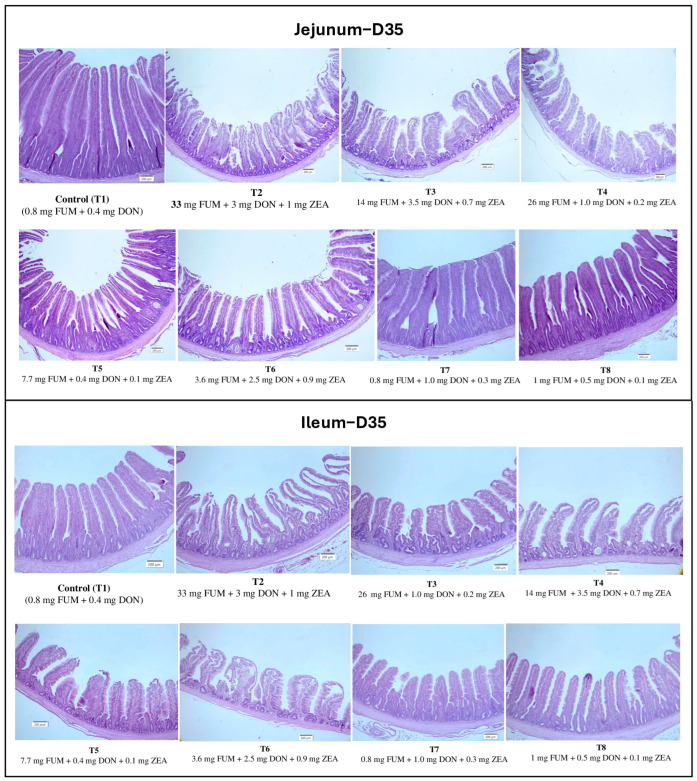
Representative hematoxylin and eosin-stained images of jejunum and ileum on d35.

On d21 and d35, birds in groups T2, T3, T4, and T5 had significantly shorter villi lengths compared to the control group (T1) (*p* < 0.05); Table 3. Similar trends were observed in ileal villi length, with T2, T3, and T6 having significantly shorter villi compared to the control group (*p* < 0.01). No significant differences in crypt depth were observed in the jejunum on d21 (*p* > 0.05) or in either the jejunum or ileum on d35 (*p* > 0.05). Mild to moderate intestinal lesions were observed on d21 and d35, characterized by broken villi at the tips and significant ulceration on the surface of both the jejunum and ileum (Figure 1).

**Figure 2:**
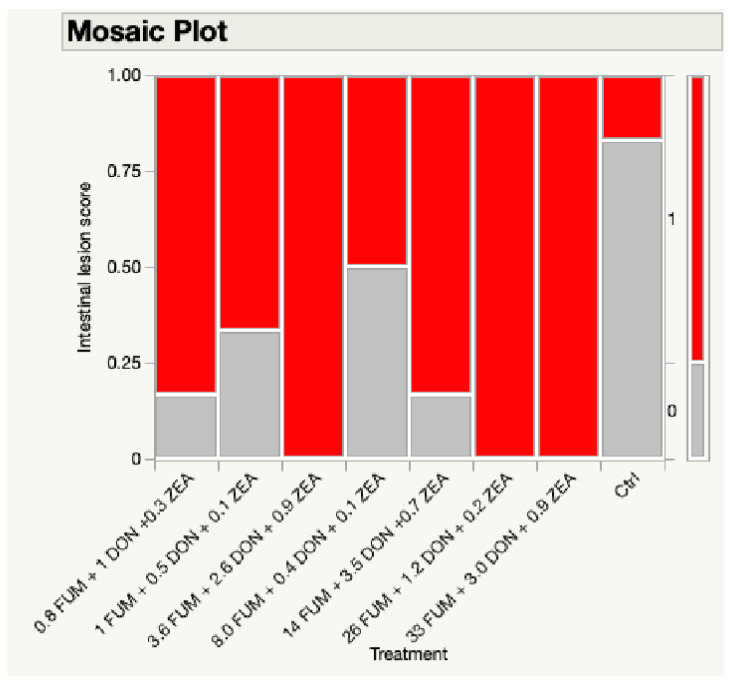
Effect of combined doses of mycotoxins on intestinal lesion score. Three birds from each pen were scored for intestinal lesions on d35, using a 0–3 scale: 0 being normal, 1 indicating a mild mucus covering the small intestine, 2 indicating a necrotic small intestinal mucosa, and 3 indicating sloughed cells and blood in the small intestinal mucosa and contents. Grey bar: lesion score of 0; red bar: lesion score of 1; lesion scores were analyzed with Chi-squared test (*p* < 0.05).

On d35, there were significant treatment effects on the intestinal lesion score (*p* < 0.05) (Figure 2). Birds in the treatment groups T2, T3, and T4 had severe lesions with lesion scores between two and three. Birds in lower mycotoxin groups T7 and T8 had less severe lesions, scoring between zero and one.

### 2.4. Effect of Combined Doses of Mycotoxins on Jejunal Tight Junction Protein mRNA Expression

**Figure 3:**
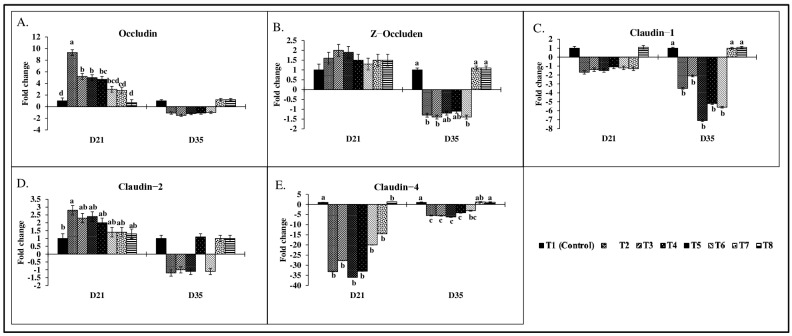
Effect of combined doses of mycotoxins on jejunal tight junction protein mRNA expression. All the mean values represent fold changes of up- and downregulated gene expression compared to the control group. Means (+SEM) with no common superscript differ significantly (*p* < 0.05) (*n* = 6). Relative gene expression levels are shown for the jejunum (**A**): Occludin (**B**): Z−occluden (**C**): Claudin−1 (**D**): Claudin−2 (**E**): Claudin−4.

There were significant (*p* < 0.05) treatment effects on the jejunal tight junction proteins mRNA transcription level on d21 and d35 (Figure 3). On d21, birds in the T2 group showed a maximum of a 9.3-fold increase in occludin mRNA transcription, compared to the control group (*p* < 0.05), whereas the T3, T4, and T5 groups had an approximately 5.0-fold increase in occludin transcription (Figure 3A) compared to the control group (*p* < 0.05). Groups T7 and T8, where the FUM+DON+ZEA concentrations were ≤1 mg/kg diet, had comparable occludin mRNA expression compared to the control group. On d21, no significant differences were observed in Z-occluden mRNA transcription (Figure 3B) between treatment groups. However, on d35, Z-occluden mRNA transcription was significantly downregulated in the T2, T3, and T6 groups compared to the control group (*p* = 0.05).

On d21, a similar trend was observed in Claudin-1 mRNA transcription (Figure 3C), but on d35, Claudin-1 transcription was significantly downregulated (*p* < 0.05) in groups T2 (3.5-fold), T3 (2.1-fold), T4 (7.1-fold), T5 (5.2-fold), and T6 (5.6-fold). On d21, Claudin-2 mRNA expression (Figure 3D) was significantly increased in T2 (2.8-fold) (*p* < 0.05), but no significant differences were observed on d35 across all treatment groups (*p* > 0.05). Claudin-4 (Figure 3E) was significantly downregulated across all treatment groups on d21, with T2 groups showing a maximum of a 33-fold reduction (*p* < 0.05). On d35, Claudin-4 mRNA expression had significantly decreased in the T2, T3, T4, T5, and T6 groups, but groups T7 and T8 had comparable Claudin-4 mRNA transcription to the control groups (*p* < 0.05).

### 2.5. Effect of Combined Doses of Mycotoxins on Splenic Macrophage Nitric Oxide Assay

**Figure 4:**
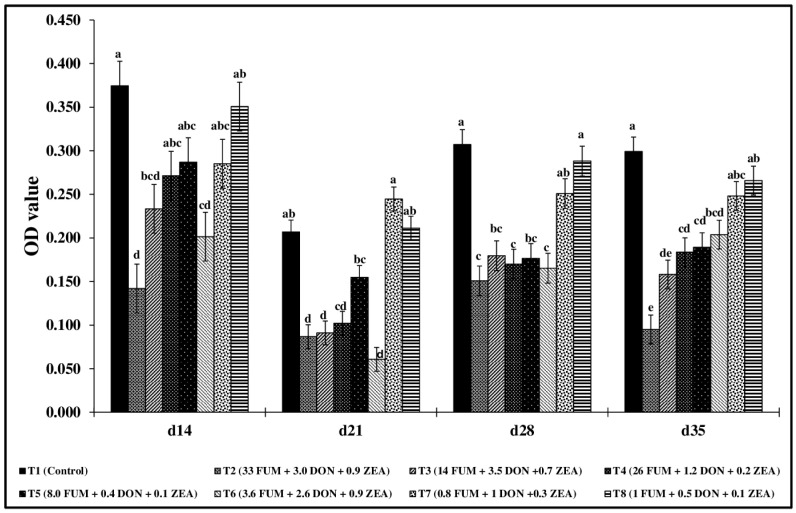
Effect of combined doses of mycotoxins on splenic macrophage nitric oxide assay. On d14, d21, d28, and d35, the splenocyte MNCs (1 × 10^5^ cells) were isolated and stimulated in vitro with 10 µg/mL of LPS for 48 h, and nitric oxide concentration was measured in the culture supernatant using the Griess assay. Means (+SEM) with no common superscript differ significantly (*p* < 0.05) (*n* = 6).

Significant reductions (*p* < 0.05) in macrophage nitric oxide production were observed across all treatment groups from d14 to d35 (Figure 4). The T2 group, exposed to the highest concentration of mycotoxins, had significantly lower nitric oxide production across all points of study. On d14, nitric oxide production in T2, T3, and T6 decreased by 62.1%, 37.8%, and 46.1%, respectively, compared to the control group (*p* < 0.05). Similar trends were observed in d21, d28, and d35, with significant reductions in nitric oxide production across treatment groups compared to the control.

### 2.6. Effect of Combined Doses of Mycotoxins on Total IgA Quantification in Bile by ELISA

**Figure 5:**
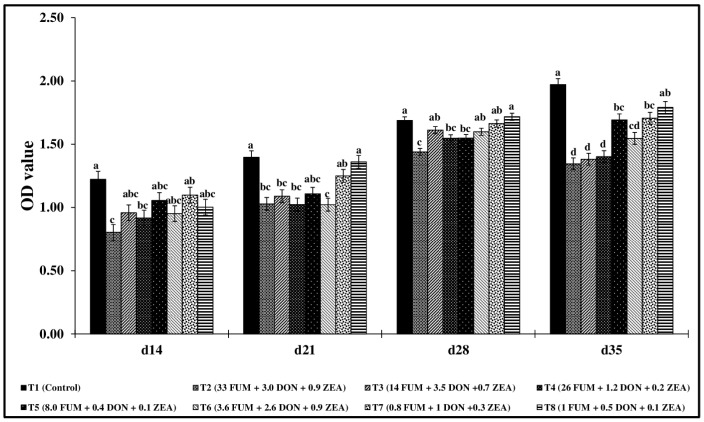
Effect of combined doses of mycotoxins on total bile IgA. Bile samples were analyzed for total IgA at d14, d21, d28, and d35 and expressed as optical density values. Means (+SEM) with no common superscript differ significantly (*p* < 0.05) (*n* = 6).

A significant decrease in total bile IgA production was observed across the highest mycotoxin treatment groups and at all time points in this study compared to the control group T1 (*p* < 0.05) (Figure 5). The T2 group with the highest mycotoxin concentration had the significantly lowest total bile IgA production on d14, d21, d28, and d35, with maximum reduction observed on d14 and d35 compared to the control T1 (*p* < 0.05). The treatment groups T7 and T8 had comparable IgA levels at all the time points compared to the control T1 (*p* > 0.05).

### 2.7. Effect of Combined Doses of Mycotoxins on Cecal Tonsils CD4+ and CD8+ T Lymphocytes and CD4+CD25+ T Regulatory Cell Percentages

A significant treatment effect was observed on cecal tonsils CD4+ and CD8+ percentages across all time points (*p* < 0.05) (Table 4). On d21, CD4+ T-cell percentages were significantly lower in all treatment groups, with the maximum decrease observed in T2 groups (69% reduction) and T3 (76%) compared to the control (T1) (*p* < 0.05). A similar trend was observed in CD8+ T-cell percentages compared to the control group (*p* < 0.01). However, the CD8+/CD4+ ratio did not show significant differences in d14, d21, and d28, but the ratio was increased in d35 compared to the control group (*p* < 0.05). On d35, Tregs percentage significantly increased in T2, T3, T4, T5, and T6 compared to the control group (*p* < 0.05).

### 2.8. Effect of Combined Doses of Mycotoxins on Spleen CD4+ and CD8+ T Lymphocytes and CD4+CD25+ T Regulatory Cell Percentages

A significant treatment effect was observed in spleen immune cell subpopulations across all time points (*p* < 0.05) (Table 5). On d14, birds in the T2 group had a lower CD4+ percentage (69%) in the spleen compared to the control group (*p* < 0.05). CD8+ percentages were significantly higher in the T6 group (86%) compared to the control (*p* < 0.05). The CD8+/CD4+ ratio was significantly higher in the T4 group compared to the control group (*p* < 0.05). A similar trend was observed on d21, with birds in the T2 group having a lower CD4+ percentage (74%) and a significantly higher CD8+ percentage (81%) in the spleen compared to the control group (*p* < 0.05), resulting in a significantly higher CD8+/CD4+ ratio (*p* < 0.05). On d28, although the CD4+ and CD8+ percentages remained altered, the changes were less pronounced, but the CD8+/CD4+ ratio was still higher in the T3 and T4 groups compared to the control (*p* < 0.05). On d35, T5 and T3 groups had a higher CD8+ percentage compared to the control (*p* < 0.05). The CD8+/CD4+ ratio remained high in the T5 group compared to the control group (*p* < 0.05). T regulatory cells were consistently reduced across treatment groups at earlier time points, but by d35, Tregs were higher in the T2 to T6 groups compared to the control group (*p* < 0.05).

### 2.9. Effect of Combined Doses of Mycotoxins on Liver mRNA Expression

**Figure 6:**
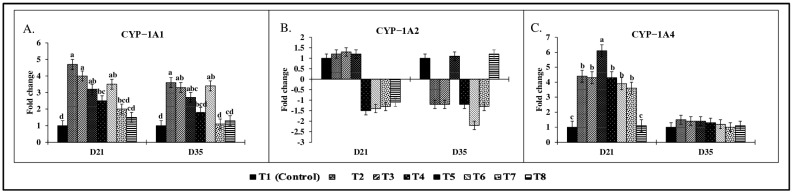
Effect of combined doses of mycotoxins on liver cytochrome mRNA expression. All the mean values represent fold changes of up- and downregulated gene expression compared to the control group. Means (+SEM) with no common superscript differ significantly (*p* < 0.05) (*n* = 6). Relative expression levels are shown for liver (**A**) CYP-1A1, (**B**) CYP-1A2, and (**C**) CYP-1A4.

**Figure 7:**
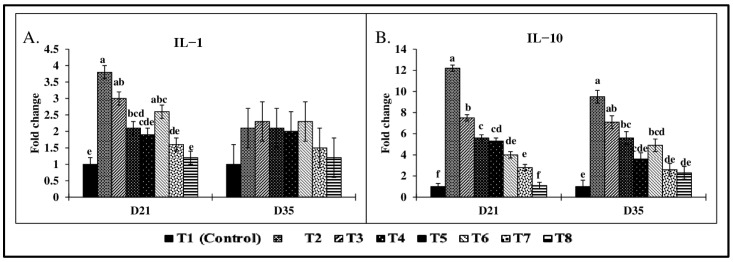
Effect of combined doses of mycotoxins on liver cytokines mRNA expression. All the mean values represent fold changes compared to the control group. Means (+SEM) with no common superscript differ significantly (*p* < 0.05) (*n* = 6). Relative expression levels are shown for liver (**A**) IL−1 and (**B**) IL−10.

There were significant (*p* < 0.05) treatment effects on liver CYP-1A1, CYP-1A4, IL-1, and IL-10 mRNA expression on both d21 and d35 (Figure 6 and Figure 7). On d21, CYP-1A1 mRNA expression (Figure 6A) in the T2 group showed a 4.7-fold increase compared to the control group. There were no significant differences observed in CYP-1A2 mRNA expression (Figure 6B) on d21 and d35 across all treatment groups (*p* > 0.05). On d21, there were significant (*p* < 0.05) increases in CYP-1A4 mRNA expression (Figure 6C) across treatment groups. Birds in the T2 and T4 groups showed significantly increased CYP-1A4 expression (4.4-fold; 6.1-fold) compared to the control group. Similarly, IL-1 expression (Figure 7A) in the T2 group was significantly increased by 3.8-fold (*p* < 0.05), while IL-10 expression (Figure 7B) was upregulated by 12.2-fold compared to the control (*p* < 0.05). A similar trend was observed on d 35, with a 9.5-fold increase in IL-10 mRNA expression and a 3.6-fold increase in CYP-1A1 expression in the T2 group compared to the control group (*p* < 0.05).

## 3. Discussion

Cereal grains used in animal diets are contaminated with various mycotoxins [48]. Among these, *Fusarium* mycotoxins, such as FUM, DON, and ZEA, have potential co-occurrence and are commonly present in broiler chicken feed [49]. Even at low concentrations, the co-occurrence of these toxins is a significant concern due to their potential synergistic effects [50]. Therefore, the objective of the study was to identify the lowest combination of FUM, DON, and ZEA that would not have a negative impact on the health and performance of broiler chickens. In the current study, chickens were exposed to different combinations of subclinical doses of FUM, DON, and ZEA to identify the dose that did not negatively affect chicken production performance and immunological status by measuring various parameters, including growth performance, ileal amino acid digestibility, intestinal morphology, T cell percentage, and CYP450 apoptosis marker.

In the present study, when the chickens were exposed to either high (33.0 mg FUM + 3.0 mg DON + 0.8 mg ZEA) or low doses of mycotoxin combinations (1.0 mg FUM + 0.5 mg DON + 0.1 mg ZEA), no significant differences in BWG were observed until d14. However, from d21 to d35, a significant reduction in BWG (13–17%) was observed in the groups exposed to the highest mycotoxin combinations, such as T2 (33 mg FUM + 3 mg DON + 0.8 mg ZEA/kg diet) and T3 (14 mg FUM + 3.5 mg DON + 0.7 mg ZEA/kg diet). In contrast, the BWG in the T7 and T8 treatment groups was comparable to the control group (T1). These results are consistent with previous research by Kubena et al. (1997) [21], who reported a 19% reduction in BWG on d21 when chickens were exposed to 300 mg FB1 + 15 mg DON per kg diet. Similar results were observed when broiler chickens were exposed to a 20 mg FUM + 1.5 mg DON/kg diet and a 20 mg FUM + 5.0 mg DON/kg diet for 21d, which led to a 6% reduction in BWG [19]. Although FCR was not significantly affected by different combinations of mycotoxins, a numerical increase in FCR was observed on days 28 and 35 across the treatment groups. A longitudinal study on broiler chickens exposed to a combination of low doses of FUM (2.6–3.5 mg/kg), DON (1.5–2.6 mg/kg), and ZEA (0.18–0.24 mg/kg) also found a significant increase in FCR [14]. The decreased BWG and increased FCR observed in this study suggest that mycotoxins may reduce nutrient digestibility and cause malabsorption in chickens, leading to decreased feed conversion into body mass.

Amino acids are essential for chicken growth performance and immune function. In this current study, there was a reduction in the digestibility of the amino acids, with aspartate and serine decreasing by 27% and 23%, respectively, in the highest DON-containing group, T3 (14 mg FUM + 3.5 mg DON + 0.7 mg ZEA/kg diet). The T6 group (3.6 mg FUM + 2.6 mg DON + 0.9 mg ZEA/kg diet) also showed a 9.6% reduction in methionine digestibility. There were notable trends for threonine (*p* = 0.06), glutamate (*p* = 0.07), and tyrosine (*p* = 0.06). However, treatment groups containing mycotoxin levels ≤ 1 mg/kg diet did not significantly affect ileal amino acid digestibility.

Mycotoxins FUM and DON have low absorption in the intestinal tract, leading to higher concentrations in the distal part of the intestinal lumen, which results in poor ileal nutrient digestibility by damaging the intestinal mucosa and disrupting transport mechanisms [43]. Awad et al. (2004) [51] demonstrated that broilers exposed to DON exhibited significantly impaired nutrient transport and amino acid absorption, primarily due to damage to the intestinal epithelium, suggesting a direct link between DON exposure and nutrient malabsorption in poultry. Methionine is essential for poultry growth and immune responses, as it plays a critical role in protein synthesis and methylation reactions [52]. Methionine deficiencies are associated with impaired growth performance and weakened immune responses due to decreased lymphocyte proliferation [53]. Therefore, the 9.6% reduction in methionine digestibility observed in this study could directly correlate with impaired protein synthesis and immunosuppression in broilers.

The T3 group showed the lowest threonine digestibility (48.2%) (*p* = 0.06), suggesting that the 14.0 FUM + 3.5 DON + 0.7 ZEA combination adversely impacted threonine absorption. Threonine is essential for mucin production, a critical component of gut health. Mucins support immune function by providing a protective barrier against pathogens [54]. The 48.2% decrease in threonine digestibility observed in T3 groups suggests that reduced threonine availability could compromise mucosal barrier function, particularly in the presence of mycotoxins [55]. Similar results were observed in growing pigs exposed to 10 mg/kg DON, which resulted in the reduced apparent ileal digestibility of indispensable amino acids lysine, threonine, tryptophan, and valine [56]. A systematic review confirms the relationship between mycotoxin exposure and reduced nutrient absorption across animal models, suggesting that these impacts are broadly relevant across species [57,58]. DON promotes intestinal inflammation and disrupts the intestinal barrier, further exacerbating nutrient malabsorption and reducing amino acid availability [59].

Despite the absence of statistical significance, T3 groups negatively affect glutamate digestibility (*p* = 0.07). This decreased glutamate may adversely impact gut epithelial cell metabolism and energy production, potentially disrupting intestinal homeostasis. Furthermore, the decreased tyrosine digestibility in the T3 group (*p* = 0.06) suggests that the 14.0 FUM + 3.5 DON + 0.7 ZEA combination alters metabolic regulation.

The significant reductions in aspartate (50.9%) and serine (52.3%) digestibility in the highest mycotoxin group (T3) could prevent the availability of these amino acids for immune and stress responses. Aspartate and serine are precursors for critical compounds involved in cellular redox balance and immune response pathways [60,61]. Aspartate and serine are necessary for synthesizing antioxidants that help mitigate oxidative stress when animals are exposed to mycotoxins [62]. Our research findings suggest that even mycotoxin doses above 1 mg/kg diet can compromise amino acid digestibility, potentially impairing growth, protein synthesis, and immune function in broilers [63].

Gut morphometric characteristics, including villi height and crypt depth, are reliable indicators of nutrient absorption and immune function [54]. In the current study, exposure to a mycotoxin above 1 mg/kg compromised gut morphology, as demonstrated by ulceration and a reduction in jejunal and ileal villi height in the T2, T3, and T4 groups. These results are consistent with findings from Cheng et al. (2018) [64], who reported similar reductions in villus height in broiler chickens exposed to 1.8 mg/kg DON + 11 AFB1 + 0.4 ZEA mycotoxins for 42 days. Awad et al. (2011) [65] also reported significant reductions in jejunal villus height when chickens were exposed to 1 mg/kg and 5 mg/kg DON in their diet, leading to decreased nutrient absorption. In the present study, the combination of 33 mg FUM + 3 mg DON + 0.8 mg ZEA/kg diet decreased the jejunal and ileal villi height by 19% in the current study, with this reduction persisting until d35. Crypt depth was also significantly reduced, suggesting that mycotoxin-induced irritation in the small intestine affects the absorption ability and mucin production [66,67], which likely contributed to the impaired growth performance observed in this study. Interestingly, in the T7 and T8 groups, where mycotoxin concentrations were lower (≤1 mg/kg diet), villi height and crypt depth were comparable to those in the control group, suggesting that the mycotoxin doses ≤ 1 mg/kg diet did not negatively affect intestinal histomorphology. These findings suggest a potential threshold below which mycotoxins do not compromise gut integrity.

Tight junction proteins are important for maintaining intestinal integrity; however, their expressions were altered in the higher mycotoxin-treated groups. On d21, the upregulation of occludin and Claudin-2 expression (pore-forming proteins) in the jejunum of T2 and T3 groups suggests that combined doses of mycotoxins compromised the gut epithelium, increasing the permeability to various solutes [68]. In contrast, the downregulation of Claudin-4, a pore-sealing protein, by 33-fold in the T2 group and 14-fold in the T7 group suggests that even chronic exposure to low doses (0.8 FUM + 1 DON + 0.3 ZEA) can result in gut damage over time. These findings align with those results reported by Bracarense et al. (2012) [69], who reported similar effects on the tight junction protein expression in piglets and broiler chickens to FUM (3 mg/kg) and DON (4–10 mg/kg) [24,35]. Alterations in the Claudin expression profile can affect paracellular transport and fluid balance, potentially leading to intestinal inflammation and disruption of the intestinal microflora [25]. Even doses as low as 3.6 FUM + 2.6 DON + 0.9 ZEA may contribute to gut inflammation, resulting in the increased loss of amino acids that support intestinal integrity and permeability, as demonstrated in this study. On the other hand, on d35, the tight junction protein expression profile in the T7 and T8 groups was relatively similar to that of the control group, suggesting that lower toxin levels may allow for the recovery of gut barrier integrity over time.

Mycotoxins are known to exert immunosuppressive effects, primarily by reducing macrophage nitric oxide (NO) production, which plays an important role in the innate immune response [70]. In the current study, the T2, T3, and T6 groups showed significantly decreased NO production until d35, indicating impaired macrophage function. These findings align with previous reports by Dresden-Osborne and Noblet (2002), Moon (1998), and Sugiyama et al. (2011) [71,72,73], which demonstrated that DON, FUM, and aflatoxin B1 reduced NO production by inhibiting inducible nitric oxide synthase (iNOS) in murine macrophages stimulated with lipopolysaccharide (LPS). This suppression of NO is likely due to the inhibition of iNOS [74], a key enzyme responsible for NO synthesis. This impairment compromises the macrophage’s antimicrobial activity and weakens immune signaling, thereby reducing the bird’s ability to fight infections [75]. Furthermore, mycotoxins inhibit the IFN-γ and TNF-α signaling pathways that activate iNOS [76]. Additionally, oxidative stress caused by mycotoxins most likely damages the macrophages, further decreasing NO production [77]. In this current study, even doses as low as 3.6 FUM + 2.6 DON + 0.9 ZEA may interfere with the L-arginine metabolic pathway [78], a critical factor for NO synthesis, ultimately resulting in decreased NO production on d35.

The intestinal mucosa not only serves as a vital site for digestion and absorption but also acts as an immune organ that defends against pathogens [79]. Mucosal immunity is primarily maintained by the production of immunoglobulin A (IgA). In this current study, total bile IgA concentrations were significantly reduced across all time points in groups exposed to higher mycotoxin concentrations, specifically T2, T3, T4, and T6, with the maximum reduction observed on d14 and d35. These results are consistent with Shanmugasundaram et al. (2023) [25], where combined FUM (3 mg/kg) + DON (4 mg/kg) decreased total IgA concentration [25]. Further, FB1 (200 mg/kg) + AFB1 (0.2 mg/kg) in the feed decreased antibody titer against the Newcastle disease virus [80], and DON (10 mg/kg) reduced antibody production against the infectious bronchitis virus [81]. In contrast, treatment groups T7 and T8 showed total IgA concentrations comparable to the control group, suggesting that lower mycotoxin doses do not negatively impact IgA production. These findings indicate that exposure to combined mycotoxins affects mucosal immunity in a dose-dependent manner. The mycotoxin doses above 1 mg/kg diet can induce oxidative stress and inflammation, potentially compromising gut integrity and increasing intestinal permeability. This impairment most likely allowed the immune system to be greatly exposed to toxins and pathogens, ultimately reducing IgA production. Additionally, mycotoxins interfere with the TGF-β signaling pathway, which is important for IgA class switching and the production of B cells [82]. In contrast, comparable IgA concentrations in T7 and T8 suggest that exposure to FUM and DON above 1 mg/kg diet concentration adversely affects mucosal immunity in broiler chickens.

In the current study, higher concentrations of combined FUM and DON resulted in a decrease in the immune cell percentages of CD4+ and CD8+ T lymphocytes in the cecal tonsils and CD4+ T cells in the spleen. Birds in the T7 and T8 treatment groups, which had the lowest mycotoxin concentration (1 mg FUM + 0.5 mg DON + 0.1 mg ZEA/kg diet), exhibited immune cell percentages similar to those of the control group, suggesting that higher doses of mycotoxin may have immunosuppressive effects. A significant decrease in the percentages of CD4+ and CD8+ T lymphocytes in the cecal tonsils was observed across mycotoxin treatment groups from T2 to T6, indicating a compromised cytotoxic immune response. These findings are consistent with Girish et al. (2010) [83], who reported that *Fusarium* mycotoxins (DON at 3.9 μg/g and ZEA at 0.7–0.8 μg/g) significantly decreased the cecal tonsil CD8+ lymphocytes in broiler chickens. DON at high doses induces leukocyte apoptosis by activating the MAPK pathway, leading to immunosuppression [84]. In contrast to the cecal tonsils CD8+ T cell percentages, the spleen CD8+ T lymphocyte significantly increased across the T2 to T7 mycotoxin treatment groups. Previous research by Peng et al. (2014) [85] showed that AFB1 (34.3–134 µg/kg) treatment in broilers increased CD8+ T cells in the spleen in a dose-response manner. However, broiler chickens exposed to T-2 toxin (0.5, 1, and 2 mg/kg) had decreased CD8+ T lymphocytes in the spleen [86]. The CD8+/CD4+ ratio in both the cecal tonsils and spleen followed a similar trend to the CD8+ T lymphocyte percentages, suggesting that mycotoxin doses above 1 mg/kg diet most likely induce dysregulation of the immune system.

Chicken T regulatory cells (Tregs) are characterized by the expression of CD4+ and CD25+ on their cell surface [87], which play a key role in immune system regulation [88], thereby preventing inflammatory responses [89]. In the present study, a significant increase in the percentage of Tregs was observed across mycotoxin-contaminated groups from T2 to T6 in both the spleen and cecal tonsils following exposure to combined mycotoxins (FUM, DON, and ZEA). The highest percentage of Tregs was observed in the T2 (33 mg FUM + 3.0 mg DON + 0.9 mg ZEA) and T3 (14 mg FUM + 3.5 mg DON + 0.7 mg ZEA) treatment groups in the spleen on d35; in contrast, Treg percentages were significantly reduced in the cecal tonsils from the T2 to T6 groups. The observed increase in Tregs in the spleen, along with the decrease in Tregs in the cecal tonsils, suggests a complex immunosuppressive effect of mycotoxin exposure. The increase in Tregs in the spleen indicates the immune system’s attempt to counteract the inflammatory response induced by mycotoxins, while the decrease in Tregs in the cecal tonsils is most likely due to an impairment in local immune regulation. This dysregulation could compromise the overall immune response, ultimately increasing the broiler chicken’s susceptibility to infectious diseases.

The increased expression of pro-inflammatory cytokine IL-1 in the T2-T6 treatment groups on d21 further supports the induction of apoptosis [29,90]. During mycotoxin exposure, even doses above 1 mg/kg diet increased the IL-1. IL-1 is known to activate hepatic apoptotic signaling pathways during stress and inflammation [91]. In contrast, the increase in the expression of IL-10, an anti-inflammatory cytokine, indicates that the immune system is attempting to modulate and control apoptosis. IL-10 is primarily produced by Tregs and plays a critical role in suppressing inflammatory responses and promoting immune homeostasis [88]. By enhancing IL-10 production, Tregs most likely limits excessive inflammation and apoptosis in response to mycotoxin exposure. However, the imbalance between pro- and anti-inflammatory cytokine expression could lead to tissue damage and cell death. In the current study, the increased expression of CYP-1A1 and CYP-1A4, along with increased IL-1 and IL-10 expression on d35, strongly suggests that apoptosis is a consequence of chronic exposure to subclinical doses of mycotoxins. Presumably, Tregs may be responding to regulate the inflammatory response through IL-10 production [92]. However, the persistent elevation of pro-inflammatory signals could reduce the protective effects of Tregs, ultimately leading to compromised overall chicken health.

In summary, this study highlights the detrimental effects of chronic exposure to combined mycotoxins in broiler chickens, even at subclinical doses. In this current study, when broiler chickens were fed mycotoxin concentrations well below the FDA-recommended guidelines, there was a significant reduction in body weight gain, disruptions in intestinal morphology, immune regulation, intestinal health, CYP-1A1, CYP-1A4, and cytokine expression. According to our findings, the concentration of mycotoxins ≤ 1 mg/kg diet (T7: 0.8 FUM + 1 DON + 0.3 ZEA and T8: 1 FUM + 0.5 DON + 0.1 ZEA) appears to be an acceptable limit at which these toxins did not negatively impact broiler chicken health. Given that mycotoxins impact amino acid digestion and intestinal health, conducting future research to modify the diet’s nutritional composition could potentially compensate for the impaired amino acid absorption. Further, including supplements that strengthen the immune system may also help reduce oxidative stress caused by mycotoxins.

## 4. Conclusions

In conclusion, the combined mycotoxin concentration above 2.5 mg/kg has shown a significant effect on the growth performance, immune response, and intestinal health of broilers. Our research findings suggest that poultry producers could benefit from not only monitoring the maximum permissible levels of mycotoxins in the chicken diet but also from implementing routine feed testing and considering the combined effects of multiple mycotoxins rather than focusing solely on individual toxins. Ultimately, such monitoring is equally important for ensuring optimal broiler chicken production performance and health, especially when raising chickens in an antibiotic-free environment that may have increased disease pressures.

## 5. Materials and Methods

### 5.1. Diet Formulation

Two strains of Fusarium, *F. graminearum* strain PH-1 and *F. verticillioides* FRC M-3125, were cultured for DON and FUM production, respectively [24]. In brief, *Fusarium* strains were individually cultured in carboxymethyl cellulose liquid media, with *F. verticillioides* for 5 days and *F. graminearum* for 7 days, after which spores were harvested. These fungal spores were then added to rice media and incubated at 28 °C for 14 days in the dark. The rice culture was freeze-dried and stored at 4 °C until the mycotoxin levels were analyzed. The rice cultures containing FUM and DON were homogenized, mixed with a portion of the basal diet, and then combined with the required amount of basal feed to make the experimental diets. The *F. graminearum* (PH-1) also produced ZEA in addition to DON. Hence, ZEA was present in all the experimental treatment groups at concentrations ranging from 0.1 to 1 mg/kg of diet. A non-medicated corn-soybean meal-based mash diet was used as the basal diet. Feed formulation and the proximate analysis of the experimental diet are given in Table 6 and Table 7. The feeding trial was conducted in two phases: (1) the starter diet (d0–d21) and (2) the grower diet (d21–d35). The final diets were tested for the actual concentrations of FUM, DON, and other major mycotoxins using LC-MS-MS (Romer Labs, Union, MO, United States), and the results are presented in Table 8. To determine apparent ileal amino acid digestibility, titanium dioxide (TiO_2_) was served as a biological marker and included in the starter diet at a 0.5% concentration in each treatment diet. TiO_2_ allows quantification of nutrient digestion by serving as a reference point for measuring the concentration of amino acids and crude protein in the ileal digesta samples.

### 5.2. Birds and Housing

A total of 960 one-day-old Ross x Ross 708 strain male broiler chicks were sourced from a commercial hatchery (Aviagen, Blairsville, GA, USA) and vaccinated at day 0 against Eimeria using CocciVAC. Prior to the experiment, the chicks were observed for any signs of illness to ensure uniform health status across the treatment groups. Chicks were weighed individually and randomly assigned to eight treatments. Treatment groups were T1 (Control), basal diet (0.8 mg FUM + 0.4 mg DON/kg diet); T2, 33.0 mg FUM + 3.0 mg DON + 0.8 mg ZEA/kg diet; T3, 14.0 mg FUM + 3.5 mg DON + 0.7 mg ZEA/kg diet; T4, 26.0 mg FUM + 1.0 mg DON + 0.2 mg ZEA/kg diet; T5, 7.7 mg FUM + 0.4 mg DON + 0.1 mg ZEA/kg diet; T6, 3.6 mg FUM + 2.5 mg DON + 0.9 mg ZEA/kg diet; T7, 0.8 mg FUM + 1.0 mg DON + 0.3 mg ZEA/kg diet; and T8, 1.0 mg FUM + 0.5 mg DON + 0.1 mg ZEA/kg diet. Each treatment was replicated across six pens, with 20 birds per pen, and the study concluded on day 35. All animal protocols were approved by the Institutional Animal Care and Use Committee at the Southern Poultry Research Group, Athens, GA (US-NE042022-54, approved on 11 March 2022). The birds were observed twice daily, and animal care procedures followed the Guide for the Care and Use of Agricultural Animals in the Research and Teaching [94]. Day-old broiler chicks were housed in 1.5 × 1.5 m floor pens with fresh pine shavings, in line with standard industry practices in North America. The pens were equipped with nipple drinkers and feeders, and environmental conditions were controlled using thermostatically regulated heaters and fans. Chicks had ad libitum access to feed and water throughout the experimental period.

### 5.3. Growth Performance

Body weight and feed intake were measured on days 0, 7, 14, 21, 28, and 35. Daily mortality rates were also recorded. BWG and average feed intake were corrected for mortality and for calculating the FCR for each pen. On d21 and d35, sampling birds from each replicate were euthanized by cervical dislocation, and spleen, cecal tonsils, jejunum, ileum, and ileal digesta samples were collected for analysis.

### 5.4. Determination of Apparent Ileal Amino Acid Digestibility

On d21, for ileal amino acid digestibility, ileal digesta were collected from the distal half of the ileum (distal to the Meckel’s diverticulum to proximal to the ileocecal junction), by flushing with distilled water into plastic containers, following cervical dislocation. Digesta from three birds per replicate were pooled, resulting in six samples per treatment (*n* = 6), which were then stored at −20 °C until further analysis. The ileal digesta samples were analyzed for DM at 105 °C for 16 h (method 934.01 AOAC, 2006). The amino acids were determined in an AA analyzer at the University of Missouri Agricultural Experiment Station Chemical Laboratories (Columbia, MO, USA) following AOAC methods [method 982.30 E (a, b, c); AOAC, 2006] [95]. Amino acid analysis was performed by hydrolyzing the samples for 24 h in 6 N HCl at 110 °C under an atmosphere of N. For methionine and cysteine, performic acid oxidation was carried out before acid hydrolysis. The amino acids in the hydrolysate were determined by cation-exchange HPLC coupled with post-column derivatization and quantitation using NIST standard reference material 2389 a [AOAC Method 982.30 E (a, b, c)] to maintain the accuracy and traceability. Nitrogen (crude protein equals nitrogen multiplied by 6.25) was determined using a Fisions 2000 model combustion analyzer (method 990.03; AOAC, 2000). The apparent ileal digestibility (AID) of crude protein (CP) and amino acid (AA) in the assay diets was calculated according to the following equation:

$$AID,\;\%=\left\{1-\left(\frac{T_{i}}{T_{o}}\times \frac{{AA}_{o}}{{AA}_{d}}\right)\right\}\times 100$$

where $T_{i}$ = titanium content in the assay diet (% DM), AI AA_o_ = AA or CP content in ileal digesta (% dry matter (DM)), AA_d_ = AA or CP content in the assay diet (% DM), and T_o_ = titanium concentration in ileal digesta (% DM).

### 5.5. Jejunal and Ileal Histomorphology

On d21 and d35, jejunal and ileal samples were collected from one bird/pen (*n* = 6) from each replication and processed for histopathology analysis as described earlier [96]. Approximately 4 cm of jejunal and ileal samples were cut proximal and distal to the Meckel’s diverticulum and stored in 10% buffered formalin. The jejunal and ileal samples were dehydrated at room temperature in a graded series of alcohols (50%, 70%, 95%, and 100% ethanol). Samples were cleared in Pro-par (Anatech, Battle Creek, MI, USA) and infiltrated with paraffin at 60 °C overnight in a tissue processor (Sakura Finetek USA, Inc., Torrance, CA, USA). Samples were cross-sectioned at 5 μm thickness and stained with hematoxylin and eosin following the standard staining procedure. The cross-sections were viewed under an Olympus BX60 brightfield microscope (Olympus Corp., Tokyo, Japan) and analyzed using CellSens Imaging software, version 4.3.1 (Olympus America, Central Valley, PA, USA) to measure villi length and crypt depth. Ten intact lamina propria villi and crypts per section and a total of 50 sections per sample were analyzed. On d35, the intestinal lesion scoring was performed based on a 0 to 3 scale as described by [97]. In brief, three birds per replicate, for a total of 18 birds per treatment, were scored for lesion scores (*n* = 6). The following scores were used in this study: (0) is normal, (1) is a slight mucus covering the small intestine, (2) is a necrotic small intestinal mucosa, and (3) is sloughed cells and blood in the small intestinal mucosa and contents.

### 5.6. Jejunal Tight Junctions and Spleen, Liver mRNA Expression

On d21 and d35, one bird per pen (*n* = 6) was euthanized by cervical dislocation. A portion of the distal jejunum (1 cm proximal to the Meckel’s diverticulum), spleen, and liver were collected in cryovials containing RNAlater^®^ (Ambion Inc., Austin, TX, USA) and stored at −80 °C until further analysis. The jejunum samples were analyzed for mRNA expression of tight junction proteins, including Claudin-1, Claudin-2, Claudin-4, occludin, and zona-occluden. The liver samples were analyzed for interleukin (IL)-1, IL-10, and cytochrome P450 (CYP) isoforms CYP1A1, CYP1A2, and CYP1A4 mRNA expression by real-time quantitative PCR. Total RNA was extracted from the spleen, liver, and jejunum using TRI reagent (Molecular Research Center, Cincinnati, OH, USA) following the manufacturer’s instructions. RNA concentration and purity were determined using a Synergy HTX multimode microplate reader (BioTek Instruments Inc., Winooski, VT, USA), using the 260/280 and 260/230 ratios. A total of 2 μg of RNA was reverse transcribed into cDNA using a cDNA synthesis kit (Promega, Madison, WI, USA). A no reverse transcriptase control was used as a non-template control to track genomic DNA contamination. mRNA expression was analyzed by real-time PCR (CFX96 Touch Real-Time System, BioRad, Hercules, CA, USA) using SYBR Green. Primer sequences and annealing temperatures are provided in Table 9. Samples were evaluated in duplicate in 96-well plates. Each well contained 5 μL of SYBR Green PCR master mix, 2 µL of RNAse-free water, 2 µL of cDNA (~600 ng/μL), 0.5 µL of forward primer (10 µM), and 0.5 µL of reverse primer (10 µM). Real-time PCR was performed with the following conditions: initial denaturation at 95 °C for 10 min (1 cycle), followed by 40 cycles of 95 °C for 15 s and 60 °C for 45 s. After each PCR run, the melting profile of each sample was assessed to confirm PCR product specificity. The housekeeping genes β-actin, glyceraldehyde-3-phosphate dehydrogenase (GAPDH), and ribosomal protein S13 (RPS13) were selected, and their stability was analyzed using Normfinder software, version 21 (Department of Molecular Medicine, Aarhus University Hospital, Denmark) as described previously [98] (Table S1). The geometric mean of GAPDH and RPS13 genes was chosen for normalizing tight junction protein data, and the geometric mean of β-actin and RPS13 was selected for normalizing liver cytokines and CYP genes because they exhibited the most stable expression among the housekeeping genes. The mRNA fold change was calculated using the 2^−∆∆Cq^ method, as previously described [99], where Cq is the threshold cycle. The fold change from the reference was calculated as ES^(40-Ct Sample)^/ER^(40-Ct Reference^), where ES and ER are the sample and reference PCR amplification efficiencies, respectively. The reference group was the control group (T1).

### 5.7. Nitric Oxide Assay

On d14, d21, d28, and d35, macrophages were collected from the spleen of one bird in each of the six replicates per treatment (*n* = 6), as described previously [100]. Briefly, a single-cell suspension from the spleen was enriched for mononuclear cells (MNCs) using density centrifugation with Histopaque (1.077 g/mL, Sigma-Aldrich, St. Louis, MO, USA) for 15 min at 400 g. The splenocyte MNCs were then washed and resuspended in complete RPMI-1640 medium (supplemented with 4% FBS, 2% chicken serum, and 1% penicillin-streptomycin) in T75 cell culture flasks and incubated at 37 °C with 5% CO2 for 24 h. The adherent cells were removed using trypsinization, washed twice with a complete medium, and then plated in triplicates in 96-well plates (250 μL of 1 × 10^5^ cells/well). The cells were stimulated with 10 μg/mL of lipopolysaccharide (LPS) from Salmonella Enteritidis (Sigma Chemicals, MO, USA) and incubated at 37 °C for 48 h. After incubation, the plates were centrifuged at 400× *g* for 10 min, and the supernatant was collected. Nitrite concentration in the supernatant was then measured using a sulfanilamide/N-(1-Naphthyl) ethylenediamine dihydrochloride solution (#R2233500, Ricca Chemical Company, Arlington, TX, USA), following the manufacturer’s instructions. The absorbance was measured at 540 nm using a Synergy HTX multimode microplate reader (BioTek, VT, USA), and the nitrite concentration in the samples was determined using a standard curve generated with known concentrations of sodium nitrite using Gen5 software, version 3.16.10 (Epoch plate reader, BioTek, VT) in the same plate as the samples.

### 5.8. Total IgA Quantification in Bile by ELISA

On d14, d21, d28, and d35, bile was collected from the gallbladder of one bird per pen in each treatment group (*n* = 6) using a 3 mL syringe with a 22 G needle and stored at –20 °C for future analysis. Total IgA levels in the bile were quantified using an indirect ELISA with a chicken IgA ELISA kit, following the manufacturer’s instructions (Bethyl Laboratories Inc., Montgomery, TX, USA, Catalog # E33-103). Briefly, 100 μL of a 1:1600 dilution of bile in SuperBlock™ (PBS) Blocking Buffer was added in duplicates to the plates and incubated for 1 h at room temperature. After washing, 100 μL of a 1:100,000 dilution of horseradish peroxidase-labeled anti-chicken IgA (Novus Biologicals, Littleton, CO, USA) in SuperBlock™ (PBS) Blocking Buffer was added to each well and incubated for 1 h at room temperature. The plates were then washed with PBS-Tween 20, and 100 μL of 3,3,5,5-tetramethylbenzidine (TMB) solution (eBioscience, San Diego, CA, USA) was added to each well. After 10 min, the reaction was stopped with 100 μL of 1 N HCl, and the optical density at 450 nm was measured using a microplate ELISA reader. IgA levels were reported as the mean optical density.

### 5.9. Spleen and Cecal Tonsil CD4+, CD8+ T Lymphocytes, and CD4+CD25+ T Regulatory Cell Percentages

On d14, d21, d28, and d35, the combined effect of FUM, DON, and ZEA on the spleen and cecal tonsil CD4+ and CD8+, and CD4+CD25+ T-regulatory cell percentages were determined by flow cytometry as described previously [92]. Briefly, single-cell suspensions from the spleen and cecal tonsils were prepared and enriched for lymphocytes using density centrifugation with Histopaque (1.077 g/mL, Sigma-Aldrich, St. Louis, MO, USA) for 15 min at 400 g. The cells were then incubated with a 1:250 dilution of fluorescent-isothiocyanate (FITC-d) conjugated mouse anti-chicken CD4+ antibody (Southern Biotech, Birmingham, AL), a 1:450 dilution of phycoerythrin-conjugated mouse anti-chicken CD8+ antibody (Southern Biotech, Birmingham, AL, USA), and a 1:200 dilution of unlabeled mouse IgG for 15 min. After incubation, unbound antibodies were removed by centrifugation. The percentages of CD4+ and CD8+ cells were measured using a flow cytometer (Guava easyCyte, Millipore, MA, USA), and the CD8+:CD4+ ratio was calculated. For the T-regulatory cell (Tregs) percentages, cells (1 × 10^6^) were incubated with 10 μg/mL of phycoerythrin-conjugated mouse anti-chicken CD25 antibody [88], 1:250 dilution of FITC-d- conjugated mouse anti-chicken CD4+ (Southern Biotechnology Associates, Birmingham, AL, USA), and a 1:200 dilution of unlabeled mouse IgG for 45 min. The unbound primary antibodies were removed by centrifugation. The percentage of CD4+, CD8+, and CD4+CD25+ cells in the spleen and cecal tonsils was determined and calculated using a flow cytometer (Guava easyCyte, Luminex, TX, USA). For each sample, 10,000 events were analyzed. Data were analyzed using GuavaSoft 4.5 software, with dead cells excluded from the analysis. Lymphocyte populations were identified based on forward and side scatter gating. The CD4+CD25+ cells in the spleen and cecal tonsils were expressed relative to the total CD4+ cells to facilitate comparison among samples. The flow cytometry sorting chart used for gating and analysis is provided as a supplementary figure (Figure S1).

### 5.10. Statistical Analysis

A one-way ANOVA (JMP Pro 16 software, version 16.2.0, Cary, NC, USA) was conducted to analyze the effects of different concentrations of combined doses of mycotoxins on the dependent variables, with *p* < 0.05 considered significantly different. The pen was considered the experimental unit. Tendency was defined as 0.05 < *p* < 0.10. When the main effects were significant (*p* < 0.05), differences between means were analyzed by Tukey’s least-square means comparison. Values are reported as means + SEM. A non-parametric test was used to analyze the lesion scores, and the Wilcoxon/Kruskal–Wallis rank sum test was used to separate the means.

## Figures and Tables

**Table 1:** Effect of combined doses of mycotoxins on body weight gain (BWG) and feed conversion ratio (FCR). BWG and mortality-adjusted FCR were calculated on days 0, 7, 14, 21, 28, and 35. Means within a row with no common superscript differ significantly (*p* < 0.05) (*n* = 6).

Days	Parameter	T1 (Control)	T2	T3	T4	T5	T6	T7	T8	SEM	*p* Value
0–7	BWG (g)	56.3	49.3	54.2	55.9	47.9	55.4	51.3	50.2	3.2	0.42
	FCR	2.33	2.47	2.33	2.5	2.63	2.32	2.33	2.49	0.16	0.43

0–14	BWG (g)	213.3	195.9	187.0	211.9	219.9	202.8	218.3	227.4	12.8	0.38
	FCR	1.74	2.03	1.89	1.71	1.89	1.79	1.73	2.02	0.13	0.44

0–21	BWG (g)	461.2 ^a^	383.1 ^b^	408.1 ^ab^	404.6 ^ab^	419.0 ^ab^	399.6 ^b^	415.8 ^ab^	444.5 ^ab^	13.7	0.01
	FCR	1.68	1.79	1.72	1.74	1.72	1.77	1.80	1.72	0.07	0.31

0–28	BWG (g)	784.4 ^a^	684.8 ^b^	742.8 ^ab^	729.4 ^ab^	748.7 ^ab^	728.1	764.8 ^ab^	782.2 ^a^	20.2	0.03
	FCR	2.41	2.79	2.58	2.59	2.56	2.51	2.42	2.45	0.11	0.26

0–35	BWG (g)	1276.3 ^a^	1120.3 ^d^	1181.7 ^cd^	1159.8 ^cd^	1200.7 ^bc^	1203.8 ^bc^	1251.5 ^ab^	1265.2 ^ab^	14.9	<0.01
	FCR	1.68	1.79	1.77	1.76	1.72	1.72	1.74	1.67	0.03	0.16

**Table 2:** Effect of combined doses of mycotoxin on apparent ileal amino acid digestibility. Means within a row with no common superscript differ significantly (*p* < 0.05) (*n* = 6).

Item	T1 (Control)	T2	T3	T4	T5	T6	T7	T8	SEM	*p*-Value
DM%	60	59.2	54.3	62.5	63.1	53.1	63.3	60.1	4.01	0.49
Nitrogen%	75.4	71.8	71.4	77.4	77.2	70.2	74.5	76.6	3.19	0.60
Indispensable amino acids, %										
Arginine	83.5	82.5	79.3	83.8	83.0	80.1	83.4	84.8	2.17	0.62
Histidine	76.9	74.6	64.6	72.2	75.7	70.2	75.1	77.9	3.42	0.16
Leucine	78.1	76.2	66.7	73.1	77.3	71.2	75.1	78.7	3.14	0.15
Isoleucine	73.4	71.3	64.4	72.3	72.7	68.0	73.0	75.7	3.55	0.44
Lysine	80.8	80.9	76.0	84.5	83.5	78.8	80.9	83.9	2.53	0.29
Methionine	86.3 ^ab^	83.1 ^abc^	80.5 ^bc^	86.6 ^ab^	87.7 ^a^	78.0 ^c^	89.1 ^a^	85.8 ^ab^	2.15	0.01
Phenylalanine	77.1	76.3	67.2	74.4	76.2	71.1	76.2	78.2	3.11	0.24
Threonine	64.9	58.7	48.2	66.0	63.8	57.3	69.1	67.0	4.69	0.06
Valine	73.9	70.0	66.5	73.9	73.8	67.9	73.8	75.2	3.62	0.58
Dispensable amino acids, %										
Alanine	76.0	73.3	64.8	71.3	75.7	72.6	74.1	78.6	3.31	0.17
Aspartate	70.0 ^a^	70.8 ^a^	50.9 ^b^	62.8 ^a^	66.3 ^a^	63.1 ^a^	69.0 ^a^	70.1 ^a^	3.99	0.02
Cysteine	54.6	61.0	54.2	65.5	62.0	51.7	62.4	64.3	4.74	0.32
Glutamate	81.8	81.5	71.2	79.0	80.3	77.1	79.9	82.0	2.50	0.07
Glycine	65.2	63.1	68.0	58.5	61.9	56.1	68.8	65.9	4.49	0.45
Proline	70.4	67.0	57.9	65.6	68.6	61.2	67.9	71.4	3.99	0.26
Serine	68.3 ^ab^	66.9 ^ab^	52.3 ^c^	61.9 ^abc^	68.8 ^ab^	58.8 ^bc^	70.3 ^ab^	72.1 ^a^	4.05	0.02
Tyrosine	74.7	74.7	65.0	72.4	70.8	63.5	76.1	77.3	3.47	0.06
Total amino acids, %	75.6	74.0	65.6	73.4	74.8	69.4	75.2	77.0	3.20	0.24

**Table 3:** Effect of combined dose of mycotoxins on jejunum and ileal histomorphology. Means within a row with no common superscript differ significantly (*p* < 0.05) (*n* = 6).

Days of Exposure	Parameter	T1 (Control)	T2	T3	T4	T5	T6	T7	T8	SEM	*p* Value
d21											
Jejunum	Villi length (µm)	926 ^a^	749.7 ^bc^	722.6 ^bc^	638.6 ^c^	686.4 ^bc^	777.3 ^abc^	806.8 ^ab^	808.2 ^ab^	58.7	0.05
Jejunum	Crypt depth (µm)	284.6	211.1	211.7	190.9	203.7	190.0	228.7	259.8	26.6	0.16
Ileum	Villi length (µm)	559.2 ^a^	431.6 ^c^	452.7 ^bc^	462.7 ^abc^	455.4 ^abc^	446.8 ^bc^	562.9 ^a^	572.1 ^a^	23	<0.01
Ileum	Crypt depth (µm)	133.1 ^a^	113.9 ^bc^	113.5 ^bc^	118.9 ^bc^	122.4 ^abc^	89.3 ^c^	146.0 ^a^	144.5 ^a^	12	<0.01
d35											
Jejunum	Villi length (µm)	1355.3 ^a^	1071.4 ^bc^	1012.1 ^c^	1135.6 ^bc^	1094.6 ^bc^	1035.2 ^bc^	1233.5 ^abc^	1252.1 ^ab^	78.6	0.042
Jejunum	Crypt depth (µm)	246.4	198.9	181.9	217.8	237.0	234.6	234.6	233.3	22.3	0.46
Ileum	Villi length (µm)	1282.0 ^a^	1045.8 ^b^	1086.2 ^ab^	1130.8 ^ab^	1111.0 ^ab^	1082.2 ^b^	1185.2 ^ab^	1213.12 ^ab^	43.9	<0.01
Ileum	Crypt depth (µm)	255.9	209.11	205.6	210.2	227.8	210.4	238.4	255.7	20.1	0.38

**Table 4:** Effect of combined doses of mycotoxins on cecal tonsils CD4+, CD8+ ratio, and Tregs percentage. Flow cytometry was used to analyze the percentages of CD4+, CD8+ T, and CD4+CD25+ T regulatory cells in the cecal tonsils. Means within a row with no common superscript differ significantly (*p* < 0.05) (*n* = 6).

Days of Exposure	Cecal Tonsils T Cells	T1 (Control)	T2	T3	T4	T5	T6	T7	T8	SEM	*p* Value
d14	CD4+ (%)	18.0 ^a^	12.1 ^bc^	7.7 ^cd^	6.5 ^d^	4.1 ^d^	3.2 ^d^	15.8 ^ab^	15.3 ^ab^	1.1	<0.05
	CD8+ (%)	19.9 ^a^	8.5 ^b^	9.4 ^b^	8.8 ^b^	6.4 ^b^	4.1 ^b^	18.9 ^a^	15.7 ^a^	1.4	<0.05
	CD8+/CD4+ ratio	1.1	0.7	1.2	1.3	1.5	1.3	1.2	1.0	0.2	0.28
	T regs (%)	3.7 ^a^	1.9 ^b^	1.7 ^b^	1.4 ^b^	1.4 ^b^	0.9 ^b^	1.7 ^b^	3.4 ^a^	0.3	<0.05

d21	CD4+ (%)	21.5 ^a^	6.6 ^d^	5.1 ^d^	7.6 ^cd^	8.0 ^cd^	8.2 ^cd^	12.0 ^bc^	15.4 ^b^	1.1	<0.05
	CD8+ (%)	29.6 ^a^	6.9 ^c^	7.0 ^c^	7.4 ^c^	9.6 ^bc^	9.9 ^bc^	14.1 ^bc^	17.2 ^b^	1.8	<0.05
	CD8+/CD4+ ratio	1.4	1.0	1.4	1.0	1.2	1.2	1.2	1.1	0.1	0.30
	T regs (%)	4.5 ^a^	2.6 ^cd^	2.3 ^cd^	2.1 ^cd^	2.0 ^cd^	1.5 ^d^	3.2 ^bc^	4.3 ^ab^	0.3	<0.05

d28	CD4+ (%)	24.4 ^a^	8.3 ^d^	11.1 ^cd^	15.1 ^bc^	13.7 ^bcd^	12.3 ^cd^	12.5 ^cd^	20.0 ^ab^	1.5	<0.05
	CD8+ (%)	23.3 ^a^	7.6 ^d^	10.9 ^cd^	17.6 ^ab^	18.8 ^ab^	13.4 ^bcd^	13.8 ^bc^	17.5 ^ab^	1.4	<0.05
	CD8+/CD4+ ratio	1.0	1.1	1.0	1.3	1.4	1.1	1.1	0.9	0.1	0.17
	T regs (%)	3.0 ^a^	1.2 ^b^	1.5 ^b^	1.3 ^b^	1.5 ^b^	1.2 ^b^	1.9 ^ab^	2.3 ^ab^	0.3	<0.05

d35	CD4+ (%)	20.0 ^a^	8.3 ^b^	6.7 ^bc^	10.0 ^c^	13.6 ^c^	8.2 ^c^	16.1 ^ab^	18.4 ^b^	1.2	<0.05
	CD8+ (%)	17.5 ^a^	7.9 ^ab^	10.9 ^b^	13.1 ^c^	13.3 ^c^	14.8 ^c^	14.9 ^ab^	19.0 ^a^	1.2	<0.05
	CD8+/CD4+ ratio	0.9 ^b^	1.2 ^ab^	1.7 ^ab^	1.3 ^ab^	1.0 ^b^	1.9 ^a^	1.0 ^b^	1.0 ^b^	0.2	<0.05
	T regs (%)	3.0 ^a^	0.5 ^c^	1.7 ^bc^	0.7 ^c^	0.9 ^c^	1.5 ^c^	3.2 ^a^	2.8 ^ab^	0.3	<0.05

**Table 5:** Effect of combined doses of mycotoxins on spleen CD4+, CD8+, and Tregs percentage. CD4+ and CD8+ T cells and CD4^+^CD25^+^ T regulatory cell percentages in splenocytes were analyzed by flow cytometry. Means within a row with no common superscript differ significantly (*p* < 0.05) (*n* = 6).

Days of Exposure	Spleen Parameter	T1 (Control)	T2	T3	T4	T5	T6	T7	T8	SEM	*p* Value
d14	CD4+ (%)	16.7 ^a^	5.2 ^c^	6.8 ^bc^	6.2 ^bc^	8.4 ^b^	8.0 ^b^	8.5 ^b^	18.7 ^a^	0.5	<0.05
	CD8+ (%)	14.4 ^b^	22.0 ^a^	24.5 ^a^	24.8 ^a^	14.9 ^b^	26.8 ^a^	15.8 ^b^	14.6 ^b^	1.1	<0.05
	CD8+/CD4 ratio	0.9 ^e^	2.7 ^bcd^	3.7 ^ab^	4.1 ^a^	2.9 ^bc^	3.5 ^ab^	1.9 ^cde^	1.6 ^de^	0.3	<0.05
	T regs (%)	3.3 ^a^	1.5 ^c^	1.9 ^bc^	1.0 ^c^	1.4 ^c^	1.4 ^c^	1.4 ^c^	2.7 ^ab^	0.2	<0.05

d21	CD4+ (%)	17.3 ^a^	4.5 ^c^	9.0 ^b^	9.8 ^b^	8.7 ^b^	7.1 ^bc^	7.9 ^b^	17.4 ^a^	0.6	<0.05
	CD8+ (%)	17.0 ^bc^	30.8 ^a^	25.9 ^ab^	26.3 ^ab^	20.9 ^abc^	24.6 ^abc^	25.7 ^abc^	15.7 ^c^	2.2	<0.05
	CD8+/CD4 ratio	1.0 ^c^	7.5 ^a^	3.0 ^bc^	2.8 ^bc^	2.5 ^bc^	3.9 ^b^	3.4 ^b^	0.9 ^c^	0.5	<0.05
	T regs (%)	3.5 ^a^	1.8 ^b^	1.8 ^b^	1.7 ^b^	1.4 ^b^	1.2 ^b^	2.0 ^b^	3.3 ^a^	0.3	<0.05

d28	CD4+ (%)	14.9 ^a^	5.6 ^b^	5.6 ^b^	6.1 ^b^	5.7 ^b^	5.5 ^b^	6.2 ^b^	15.7 ^a^	0.7	<0.05
	CD8+ (%)	12.9	10.6	13.7	14.8	11.0	11.3	11.5	9.7	1.2	0.07
	CD8+/CD4 ratio	0.9 ^b^	2.0 ^a^	2.5 ^a^	2.4 ^a^	2.0 ^a^	2.1 ^a^	1.9 ^a^	0.6 ^b^	0.2	<0.05
	T regs (%)	3.8 ^a^	1.0 ^b^	1.6 ^b^	1.0 ^b^	0.6 ^b^	0.9 ^b^	1.3 ^b^	3.9 ^a^	0.3	<0.05

d35	CD4+ (%)	14.7 ^a^	7.1 ^cd^	6.9 ^cd^	10.9 ^b^	5.1 ^d^	9.8 ^bc^	12.3 ^ab^	11.8 ^ab^	0.8	<0.05
	CD8+ (%)	21.8 ^b^	25.5 ^ab^	30.1 ^a^	28.3 ^ab^	30.6 ^a^	26.6 ^ab^	29.7 ^ab^	19.5 ^b^	2.3	<0.05
	CD8+/CD4 ratio	1.5 ^c^	4.0 ^abc^	4.5 ^ab^	2.7 ^bc^	6.8 ^a^	2.8 ^bc^	2.6 ^bc^	1.8 ^bc^	0.7	<0.05
	T regs (%)	3.3 ^de^	7.2 ^abc^	9.0 ^a^	8.1 ^ab^	6.0 ^bcd^	5.1 ^cde^	3.8 ^de^	3.0 ^e^	0.7	<0.05

**Table 6:** Ingredient and nutrient composition of the basal diet (as-fed basis). Nutrients, vitamins, and minerals were provided to meet or exceed the value described in the NRC, a standard reference diet for chickens [93].

Ingredient	Starter (%)
Corn	56.29
Soybean meal, 48% CP	37.87
Soybean oil	2.18
Dicalcium phosphate	1.48
Calcium carbonate	0.91
Sodium chloride	0.40
MHA	0.37
L-lysine	0.21
Trace mineral premix ^1^	0.10
Choline chloride (60%)	0.07
L-threonine	0.06
Vitamin premix ^2^	0.05
Phytase (500 FTU)	0.01

^1^ Supplied per kilogram of diet: Mn, 107.2 mg; Zn, 85.6 mg; Mg, 21.44 mg; Fe, 21.04; Cu, 3.2 mg; I, 0.8 mg; Se, 0.32 mg. ^2^ Supplied per kilogram of diet: vitamin A, 5.511 IU; vitamin D3, 1.102 ICU; vitamin E, 11.02 IU; vitamin B12, 0.01 mg; biotin, 0.11 mg; menadione, 1.1 mg; thiamine, 2.21 mg; riboflavin, 4.41 mg; d-pantothenic acid, 11.02 mg; vitamin B6, 2.21 mg; niacin, 44.09 mg; folic acid, 0.55 mg; choline, 191.36 mg.

**Table 7:** Proximate analysis of starter and grower diet.

Diet	Treatment	Crude Protein (%)	Fat (%)	Moisture (%)	Crude Fiber (%)	Ash (%)	Starch (%)
Starter diet (d0–d21)							
	T1	20.42	6.10	11.79	3.64	6.74	36.89
	T2	20.31	5.92	11.99	4.07	6.60	36.67
	T3	20.63	5.69	12.11	3.74	6.10	37.73
	T4	20.75	6.04	11.56	3.70	7.12	35.92
	T5	20.44	6.00	11.78	3.67	6.84	37.33
	T6	21.07	6.08	11.69	3.79	6.88	36.63
	T7	19.81	5.96	12.03	3.91	6.00	37.18
	T8	19.83	6.20	11.73	3.84	6.39	36.95
Grower diet (d21–d35)							
	T1	18.65	6.34	11.83	3.34	6.65	40.04
	T2	18.98	5.81	12.27	3.18	6.06	39.54
	T3	18.73	5.87	11.90	3.10	6.31	42.08
	T4	19.39	5.78	12.23	3.02	6.13	40.52
	T5	19.19	5.81	12.27	3.17	6.05	40.07
	T6	19.35	5.49	12.38	3.04	6.17	39.63
	T7	19.15	5.62	12.31	2.86	6.24	40.38
	T8	20.24	5.84	12.34	2.97	6.5	39.52

**Table 8:** Analyzed mycotoxin concentrations in experimental diets by LC-MS-MS.

Treatment	Total Fumonisins (FUM) (FB1 + FB2 + FB3) (mg/kg)	FB1 (mg/kg)	DON (mg/kg)	ZEA (mg/kg)
T1 (Control)	0.8	0.6	0.4	<LOD *
T2	33.0	21.0	3.0	0.8
T3	14.0	9.0	3.5	0.7
T4	26.0	17.0	1.0	0.2
T5	7.7	5.0	0.4	0.1
T6	3.6	2.0	2.5	0.9
T7	0.8	0.6	1.0	0.3
T8	1	0.8	0.5	0.1

* Limit of detection (LOD). Representative feed samples from treatments T1 to T8 were analyzed for fumonisins (FB), deoxynivalenol (DON), and zearalenone (ZEA) concentrations using LC-MS/MS. The LOD for mycotoxin analysis in feed samples by LC-MS/MS were as follows: aflatoxin B1-1.3 ppb, aflatoxin B2-1.2 PPB, aflatoxin G1-1.1 ppb, aflatoxin G2-1.6 ppb, and ochratoxin A-1.1 ppb. For acetyl DON, fusarenon-X, nivalenol, T-2 toxin, HT-2 toxin, neosolaniol, and diacetoxyscirpenol, the LOD was set at 100 ppb. Mycotoxin concentrations below the detection limits in feed samples are not presented in the table.

**Table 9:** The sequence of primers and real-time quantitative PCR annealing temperatures of the primers used for relative gene expression analysis. F, forward primer; R, reverse primer.

Gene	Primer Sequence (5′-3′)	Annealing Temperature in °C	References
GAPDH	F-GAGGGTAGTGAAGGCTGCTG R-CCACAACACGGTTGCTGTAT	57.4	NM_001303179.1
β-actin	F-GACTGCTGCTGACACCTTCA R-ACCGGACTGTTACCAACACC	57.4	NM_001303173.1
Ribosomal protein S-13 (RPS-13)	F-CAAGAAGGCTGTTGCTGTTCG R-GGCAGAAGCTGTCGATGATT	57.0	[100] NM_001001783.2
IL-1	F-TCCTCCAGCCAGAAAGTGA R-CAGGCGGTAGAAGATGAAGC	57.5	[101] Y15006.1
IL-10	F-CATCTCTGGGCCTGAA R-CGTCTCCTTGATCTGCTTGATG	57.5	[101] NM_001004414.4
CYP 1A1	F-AGATCTGGAAGGACCCCTCC R-TAGGAGGCCAGCTGATTCCT	57.0	NM_205147.2
CYP 1A2	F-GCTTTGACACCGTGACAACC R-GTCTGCCTTCTTGCCATCCT	57.0	NM_205146.3
CYP 1A4	F-CAGAACGCCCTGAAGACCTT R-CAAGGCAGCGTACATCATGC	55.4	X99453.1
Claudin-1	F-CATACTCCTGGGTCTGGTTGGT R-GACAGCCATCCGCATCTTCT	55.0	[54] NM_001013611.2
Claudin-2	F-CCTGCTCACCCTCATIGGAG R-GCTGAACTCACTCTTGGGCT	55.0	[102] NM_001277622.1
Claudin-4	F-GAAGCGCTGAACCGATACCA R-TGCTTCTGTGCCTCAGTTTCC	57.0	[103] AY435420.1
Occludin	F-GCCTTTTGCTTCATCGCTTCC R-AACAATGATTAAAGCAAAAG	57.0	[104] NM_205128.1
Zona-occluden	F-TGTAGCCACAGCAAGAGGTG R-CTGGAATGGCTCCTIGTGGT	56.0	[105] XM_046925212.1

## Data Availability

The original contributions presented in this study are included in the article/Supplementary Material. Further inquiries can be directed to the corresponding author(s).

## Supplementary Materials

The following supporting information can be downloaded at https://www.mdpi.com/article/10.3390/toxins17010016/s1. Figure S1: Flow cytometry sorting analysis of CD4+ and CD8+ gating strategy; Table S1: NormFinder results for reference gene stability for jejunum and liver on d21 and d35.
