# Supplementary material for: Exposure to Subclinical Doses of Fumonisins, Deoxynivalenol, and Zearalenone Affects Immune Response, Amino Acid Digestibility, and Intestinal Morphology in Broiler Chickens
Source: Toxins (Basel). 2025 Jan 1;17(1):16. doi: 10.3390/toxins17010016 (PMC11769399; doi:10.3390/toxins17010016)
Supplement: Supplementary file 1 [file toxins-17-00016-s001.zip › toxins-3340080-supplementary.pdf]

Supplementary Materials: Exposure to Subclinical Doses of Fumonisins, Deoxynivalenol, and Zearalenone Affects Immune Response, Amino Acid Di-gestibility, and Intestinal Morphology in Broiler Chickens

Revathi Shanmugasundaram [1,*], Laharika Kappari [2], Mohammad Pilewar [2], Matthew K. Jones [3], Oluyinka A. Olukosi [2], Anthony Pokoo-Aikins [1], Todd J. Applegate [2] and Anthony E. Glenn [1]

Table S1. NormFinder results for reference gene stability for jejunum and liver on d21 and d35.

Gene	Jejunum		Liver	
	d21	d35	d21	d35
GAPDH & RPS13	0.020	0.007	0.010	0.016
GAPDH	0.020	0.007	0.011	0.019
RPS-13	0.029	0.010	0.015	0.021
β-actin	0.032	0.012	0.023	0.025

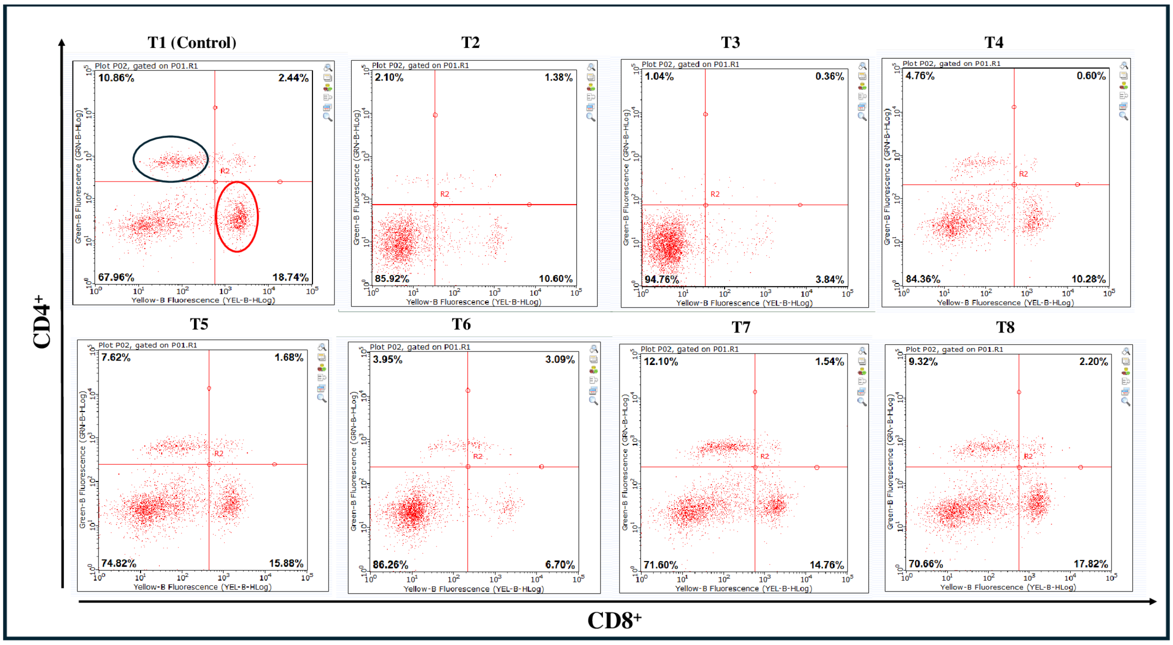

Figure S1. Flow cytometry sorting analysis of CD^{4+} and CD^{8+} gating strategy. Representative dot plot images of cecal tonsils CD^{4+} and CD^{8+} percentage on d21. Upper left quadrant was positive for CD^{4+}, Lower right quadrant was positive for CD^{8+}.
